# BOPPPS-based teaching combined with narrative pedagogy and curriculum ideology and politics in clinical nursing in gastrointestinal surgery: a quasi-experimental study

**DOI:** 10.3389/fmed.2026.1810668

**Published:** 2026-06-03

**Authors:** Xiaobei Liu, Liying Ren, Xuehong Chen

**Affiliations:** 1 School of Nursing, Anqing Medical College, Anqing, China; 2 Department of General Surgery, Anqing Municipal Hospital, Anqing, China

**Keywords:** BOPPPS teaching model, clinical teaching, curriculum ideological and political, gastrointestinal surgery, narrative education, nursing student

## Abstract

**Introduction:**

National educational policies emphasize integrating ideological and political education into curricula to enhance talent development. Clinical practice is vital in nursing education for shaping professional competencies. This study aimed to evaluate the effectiveness of a novel teaching model that integrates the Bridge-In, Objective, Pre-assessment, Participatory Learning, Post-assessment, and Summary (BOPPPS) framework with a narrative-based approach for clinical nursing education in gastrointestinal surgery. This combined model incorporates ideological-political elements to address the needs of this complex discipline, which requires innovative pedagogical methods.

**Methods:**

Ninety-two nursing students undergoing clinical training in gastrointestinal surgery from March 2023 to October 2024 were divided into a control group (*n* = 45) receiving traditional instruction and an experimental group (*n* = 47) receiving the combined BOPPPS and narrative-based approach. Outcomes were assessed using the Professional Identity Questionnaire for Nursing Students, the Nurse Core Competence Scale, clinical practice performance evaluations, and a teaching satisfaction survey.

**Results:**

The experimental group achieved significantly higher scores across all measured domains, including professional identity, core competencies, clinical performance, and teaching satisfaction, as well as in total scores compared to the control group (all *p* < 0.001). The Cohen’s d effect sizes ranged from 0.465 to 2.067, with partial *η*^2^ ranging from 0.462 to 0.970, all indicating moderate to large effects.

**Conclusion:**

The integrated BOPPPS and narrative-based teaching model significantly improved nursing students’ professional identity, core competence, clinical performance, and satisfaction, offering an innovative and effective strategy for enhancing clinical nursing education.

## 1 Background

According to the *Guiding Outline for the Construction of Curriculum Ideology and Politics in Colleges and Universities*, the Ministry of Education emphasizes the need to firmly establish the central role of talent training and to continuously improve the systems of curriculum ideology and politics, teaching, and content in order to build a high-level talent training framework (1). Clinical practice represents a critical stage in nursing education, serving as an essential teaching component for cultivating students’ professional competence and playing a decisive role in shaping their professional quality. The BOPPPS teaching model consists of six components: Bridge-in, Objective, Pre-test, Participatory learning, Post-test, and Summary (2). It emphasizes objective-oriented and student-centered teaching, aiming to fully engage nursing students, guide active exploration, foster teamwork, and enhance clinical practice skills and professional competence (3).

Narrative education is grounded in narratology theory (4) and constructivist learning theory (5). Its core principle is to facilitate knowledge internalization and support value formation through the narration, interpretation, and reconstruction of stories. This approach allows learners to engage in emotional experiences and meaning-making (6). Narratology suggests that “storytelling is a fundamental way humans understand the world” (4). Constructivist learning theory emphasizes that learners develop knowledge systems through active participation and interactive experiences (5). Together, these theories provide the foundation for narrative education. In nursing education, American medical educator Rita Charon (7) contributed an applied perspective, proposing that medical practice is inherently narrative. Both patient illness experiences and healthcare provider experiences serve as central material. Later studies have identified three functions of narrative pedagogy in nursing: supporting humanistic care and empathy in nursing students through patient narratives (8); reinforcing knowledge application and clinical reasoning via clinical case narratives (9); and promoting professional identity and value formation through teacher-student narrative interactions (10).

Gastrointestinal surgery is a highly practical discipline, and clinical nursing education faces significant challenges, including a wide variety of diseases, extensive knowledge requirements, and considerable teaching difficulties (11). Therefore, innovating teaching methods is particularly important. Integrating the principles of curriculum ideology and politics provides a clear direction for teaching reform, while combining the BOPPPS teaching model with narrative education offers an effective approach to implementing these principles. By incorporating real cases and emotional experiences into teaching, nursing students’ professional knowledge and skills can be enhanced, while their humanistic care and professional responsibility are further cultivated (12). In this study, based on the concepts of curriculum ideology and politics, the BOPPPS model and narrative education method were applied to clinical nursing teaching in gastrointestinal surgery to explore an innovative teaching approach, providing valuable references for the cultivation of nursing professionals.

## 2 Methods

### 2.1 Research object

In this study, 92 nursing students who completed clinical practice in Gastrointestinal Surgery at Anqing Municipal Hospital from March 2023 to October 2024 were selected as research participants. Inclusion criteria were: full-time nursing college students who had completed their theoretical coursework; entering the clinical practice stage in gastrointestinal surgery for the first time; and willingness to participate in the study. Exclusion criteria were: inability to complete the practice for various reasons; failure to comply with the study’s regulations; or not having completed theoretical or operational examinations. Based on the practice period, 45 nursing students who practiced from March 2023 to December 2023 were assigned to the control group, and 47 students who practiced from January 2024 to October 2024 were assigned to the experimental group. In the control group, there were 3 males and 42 females, aged 20–23 years (mean 21.42 ± 0.76), with pre-practice theoretical examination scores in surgical nursing ranging from 72 to 88 points (mean 78.16 ± 3.47). In the experimental group, there were 2 males and 45 females, aged 20–23 years (mean 21.34 ± 0.71), with pre-practice theoretical examination scores ranging from 70 to 89 points (mean 78.15 ± 3.89).

Considering the practical context of clinical nursing teaching in gastrointestinal surgery and its feasibility, several factors were taken into account. First, clinical nursing internships in gastrointestinal surgery follow a standardized rotational schedule, which makes randomized group allocation across different batches of nursing students impractical. Second, both groups of students completed the same university curriculum prior to entering the department. No statistically significant differences were observed between groups in terms of gender, age, or theoretical scores (*p* > 0.05). Third, teaching conditions, including instructors, facilities, and case resources, were consistent across groups, reducing the likelihood of cross-contamination. Fourth, during the study period, clinical nursing protocols, the internship syllabus, and teaching assessment standards for gastrointestinal surgery strictly adhered to the uniform requirements of the affiliated university and hospital, without adjustments or updates. On this basis, a quasi-experimental sequential cohort design was adopted (13, 14). All participating nursing students were verbally informed of the study’s objectives, intervention measures and data collection purposes, and all agreed to participate voluntarily.

### 2.2 Teaching methods

#### 2.2.1 Control group

The control group was taught using the traditional clinical nursing teaching method, with a practice duration of 4 weeks. On the first day of week 1, the department’s chief instructor introduced the department environment, rules, and regulations, and assigned one-to-one teaching instructors, enabling the nursing students to understand their job responsibilities and workflow. Concentrated offline theoretical lectures were provided on Mondays, while clinical teaching instructors demonstrated routine nursing procedures and skills for common gastrointestinal surgery conditions. During this period, students reviewed basic theoretical knowledge and became familiar with the key points of nursing skills. In weeks 2 and 3, instructors demonstrated standard procedures, and students practiced nursing skills under the guidance of their instructors, who addressed students’ questions promptly. In week 4, remaining practice tasks were completed, and theoretical and practical skill examinations were administered.

#### 2.2.2 Experimental group

**Figure 1:**
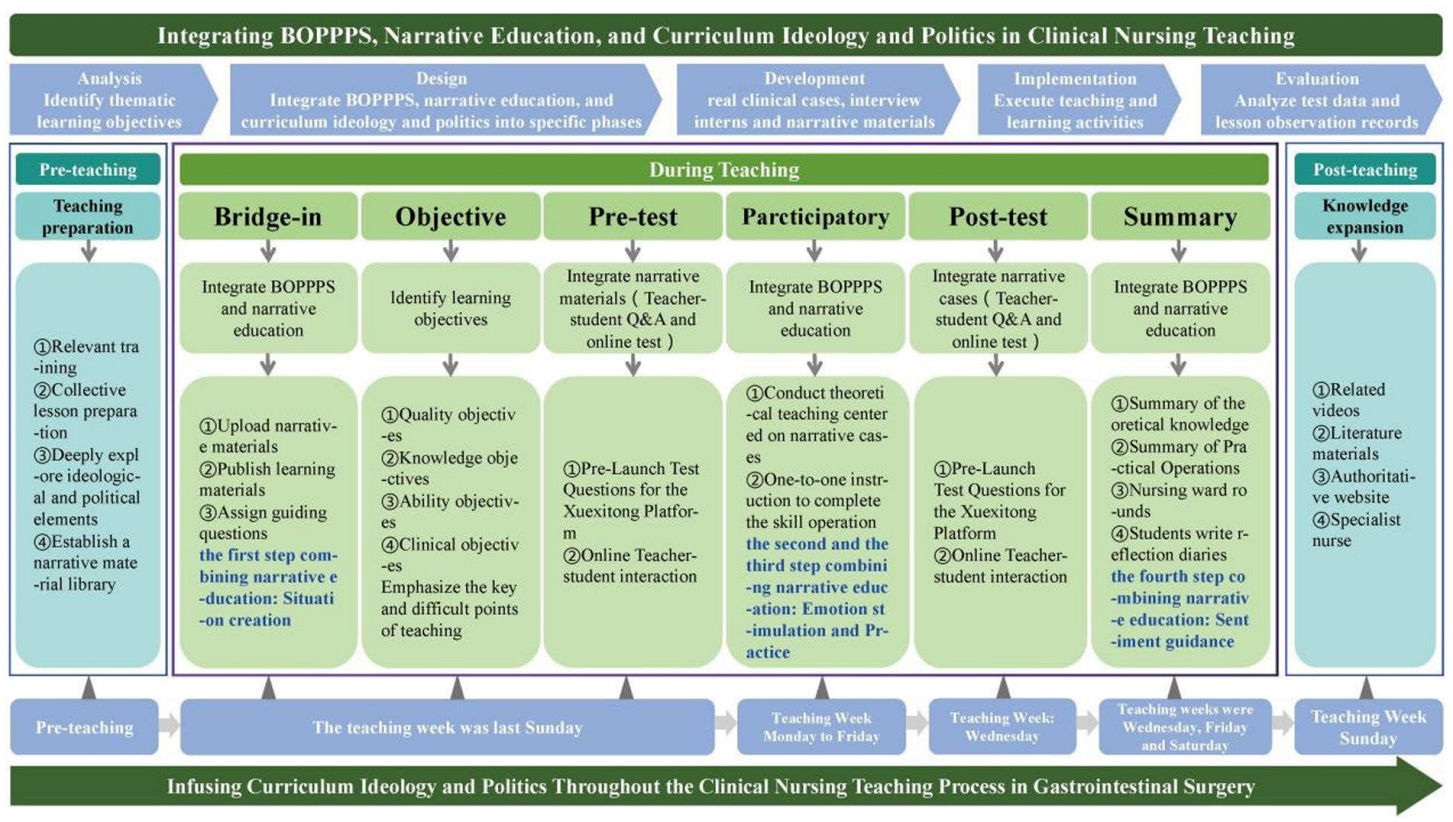
Instructional design mode for clinical nursing teaching integrating BOPPPS, narrative education, and curriculum ideology and politics.

The teaching design model was systematically developed following the stages of analysis, design, development, implementation, and evaluation, and was delivered using the BOPPPS teaching model. The narrative education method was incorporated at each stage, and the principles of curriculum ideology and politics were integrated throughout the entire clinical nursing teaching process in gastrointestinal surgery (Figure 1).

##### 2.2.2.1 Teaching preparation

(1) Standardized teacher training: The teaching team participated in training on narrative education, the BOPPPS teaching model, and the operation of the XUEXITONG smart teaching platform. Collective lesson preparation and design of teaching activities were conducted based on narrative education and the BOPPPS model, aligned with the teaching objectives and the practical work of clinical posts. The integration of curriculum ideology and politics was carefully applied, ensuring that the timing, depth, and scope were organically combined with clinical teaching content. (2) Narrative material library construction standards: The team explored ideological and political elements in clinical nursing education by collecting real clinical cases and interviewing interns (15). A narrative material library for gastrointestinal surgical nursing, incorporating curriculum ideology and politics, was established through the collection and organization of news reports, key events, popular videos, and other textual and digital resources (15). Nursing ward rounds were conducted once a week, using cases drawn from this material library. (3) On the XUEXITONG platform, classroom modules were preconfigured to include sections for material distribution, discussion, testing, interaction, and reflection sharing. Narrative materials and other resources were uploaded prior to class, and requirements for tests and reflections were established in advance.

##### 2.2.2.2 Teaching implementation

Building on the control group, the experimental group was taught using the BOPPPS model combined with narrative education, integrating curriculum ideology and politics. Guided by the *Teaching Guide for Curriculum Ideology and Politics in Nursing Majors* (16), a closed-loop teaching approach based on BOPPPS and narrative education was implemented weekly, and a synergistic system of “framework-carrier-connotation-support” was constructed. (1) BOPPPS teaching model (17): Serving as the core framework, it defined a six-step closed-loop process. This provided a standardized pathway for teaching and ensured systematic and logical instruction. (2) Narrative education (9): Acting as the carrier of teaching content, it transformed abstract nursing knowledge and ideological-political elements into concrete clinical narratives. These narratives offered perceptible and interactive material for each BOPPPS component, enhancing knowledge internalization and emotional engagement. (3) Curriculum ideology and politics (18): Providing value-oriented connotation, it embedded ideological and political elements, such as professionalism, reverence for life, and patriotism, into each teaching segment. This approach supported the integration of professional competence development with value shaping. (4) XUEXITONG platform (19): Serving as technological support, it offered an online pathway for integrating multiple components. The platform facilitated material distribution, interactive discussions, assessments, and reflective sharing. In this study, the BOPPPS framework structured the teaching process. Narrative education materials enriched the content, curriculum ideology and politics guided value orientation, and the XUEXITONG platform supported efficient implementation. Together, these elements formed an integrated teaching system for gastrointestinal surgery nursing.

Narrative education comprises four steps: creating a situation, stimulating emotion, engaging in practice, and guiding reflection (20). The implementation followed these theoretical principles: (1) Situation creation: Grounded in constructivist “situated learning theory,” this step used narrative materials from real patients to build scenarios. Students were encouraged to explore knowledge actively in contexts that resembled clinical care (15). (2) Emotion stimulation: Drawing on the “principle of emotional resonance” from narratology, this step guided students to empathize with patient experiences, supporting a deeper appreciation of nursing’s professional value (21). (3) Practice: Aligned with “learning by doing” theory, this step transformed nursing problems identified in narratives into practical tasks, facilitating the application of knowledge into skills (22). (4) Reflection guidance: Based on reflective practice theory, this step employed reflective journals to encourage thoughtful consideration of narrative experiences, promoting the internalization of professional competencies (21). The four steps of narrative education were integrated into the six components of the BOPPPS model: bridge-in, objective, pre-test, participatory learning, post-test, and summary. This approach aimed to promote students’ professional development.

Implementation of BOPPPS combined with narrative education and integrated curriculum ideology and politics proceeded as follows: (1) Bridge-in: Narrative materials were distributed via the XUEXITONG platform. Guiding questions were posted for 10 min, and students were required to complete online previews and leave comments within 24 h. Teachers summarized frequently asked questions in advance to highlight classroom focal points. (2) Pre-test/Post-test: Test questions were designed to address 40% knowledge objectives, 40% ability objectives, and 20% quality objectives. Each test included five multiple-choice questions worth 20 points each. Answers and explanations were provided immediately after the test. For questions with an error rate >30%, teachers offered centralized explanations within 24 h. (3) Participatory learning (theory): Students worked in groups of 2–3, assigning roles such as recorder and reporter. Discussions followed the sequence: “nursing problem analysis → exploration of ideological-political elements → solution construction”. Each group presented for 5 min, followed by teacher feedback that included professional correction and reinforcement of ideological-political elements. (4) Participatory learning (skills): Operational procedures and standards were clearly defined. Instructors provided one-on-one guidance using the *Quantified Skill Operation Score Sheet*, demonstrating each key point. Students advanced only after achieving independent proficiency. (5) Summary: Cases from the narrative material library were randomly selected for nursing ward rounds, following the process: “case report → problem discussion → teacher summary”. Discussions lasted at least 20 min, with teachers extracting 3–5 core knowledge points and 1–2 ideological-political points. (6) Reflection journal: A uniform template was provided, including sections for “what I learned today,” “case insights,” “career considerations,” and “areas for improvement.” Teachers offered personalized comments of at least 50 words within 48 h of submission and selected 2–3 exemplary reflections for class sharing.

Table 1 illustrates the steps of clinical nursing teaching, using “perioperative nursing of colorectal cancer patients” as the focus for week 3 practice.

**Table 1:** Steps of BOPPPS combined with narrative education method integrating curriculum ideology and politics in clinical nursing teaching.

Module	Time allocation	Implementation steps combined with narrative education	Ideological and political elements and significance
Bridge-in (B)	Sunday in week 2	A classroom was created on the XUEXITONG platform. Instructors uploaded case materials of patients with artificial stomas to the narrative material library, provided learning resources on relevant theoretical knowledge under the theme *“Rose Life,”* and designed guiding questions: (1) How can tertiary prevention and health education be implemented for colorectal cancer? (2) How should patients with colorectal cancer who experience complications from radiotherapy and chemotherapy be cared for? This link is the first step combining narrative education: Situation creation.	Professional identity: Narrative educational materials helped cultivate nursing students’ professional identity, fostering a sense of respect and reverence for the profession. Value shaping: By gaining insight into the real experiences of patients undergoing ostomy, nursing students developed the ability to respect and cherish life, thereby fostering a correct outlook on life and values.
Objective (O)	Sunday in week 2	The weekly practice objectives were released on the XUEXITONG platform, serving as both a guide for nursing students’ learning outcomes and a reference for teachers’ instructional evaluation. Quality objectives: Cultivate a patient, meticulous work attitude, and demonstrate respect, care, and compassion for patients. Knowledge objectives: Master the clinical manifestations, auxiliary examinations, and routine nursing care of colorectal cancer patients, while becoming familiar with the etiology of colorectal cancer. Ability objectives: Acquire proficiency in gastrointestinal decompression and ostomy bag replacement. Apply nursing procedures skillfully to deliver holistic care to colorectal cancer patients.	Epistemology and methodology: Clarifying the course objectives enabled nursing students to strengthen their ideals and beliefs while fostering materialist dialectics, scientific thinking, and a pragmatic work attitude. This clarity helped them focus more effectively on their studies, strive for self-improvement, and gain a clear understanding of their own learning progress.
Pre-test (P)	Sunday in week 2	In line with the narrative materials, pre-test questions, including five multiple-choice items, were released on the XUEXITONG platform: (1) What are the possible causes of colorectal cancer? (2) What are the main metastatic routes of colorectal cancer? (3) What are the earliest symptoms of colon cancer? (4) What are the main symptoms of left-sided colon cancer? (5) What are the screening methods for colon cancer? Additionally, online Q&A sessions and teacher-student interactions were conducted to reinforce learning.	Value shaping: Test questions on the causes of colorectal cancer were designed to encourage students to value health, adopt good lifestyle habits, and cultivate professional qualities rooted in respect for life. Patriotism: Test questions on colorectal cancer screening methods aimed to affirm China’s medical security policies, guide nursing students in fostering patriotism, strengthen their aspiration to contribute to national development, and inspire dedication to serving the country.
Participatory (P)	Monday of week 3	The theoretical module focused on perioperative nursing for patients with colorectal cancer. The department’s lead instructor explained and critically analyzed the narrative materials provided on the XUEXITONG platform, addressed students’ questions, and targeted key learning challenges. In response to the guiding questions in the narratives, and drawing on students’ pre-class reflections, students engaged in small-group discussions (2–3 students per group). They were required to fully explore the ideological and political elements embedded in the materials, particularly humanistic care and empathy. Faculty provided heuristic guidance throughout, and each group presented its discussion outcomes. This link is the second step combining narrative education: Emotion stimulation.	Cultural literacy: Using the story of Bian Que. and his three brothers, the concept of tertiary prevention was analyzed, highlighting China’s profound cultural heritage and rich philosophical insights while promoting the national spirit. Professional ethics: Through discussions on the complications of radiotherapy and chemotherapy, students learned to empathize with patients, cultivate patience, attentiveness, responsibility, and care, and embody the spirit of respecting humanity and cherishing life. Scientific spirit: Group discussions fostered the scientific spirit of collaboration and collective inquiry among nursing students.
	Tuesday and Wednesday of week 3	In one-to-one instruction, nursing students were guided through practical training in perioperative care for colorectal cancer patients. The practice content included: Preoperative skills: enema, indwelling catheterization, gastric tube placement, and vaginal irrigation. Postoperative skills: early mobilization, ankle pump exercises, drainage tube care, gastrointestinal decompression care, and warm-water sitz baths. Nutritional support: nursing management of enteral and parenteral nutrition. Basic clinical operations: intravenous infusion, intravenous transfusion, intramuscular injection, intradermal injection, subcutaneous injection, oral care, oxygen therapy, and management of complications. Stoma care: nursing of stomas and replacement of ostomy bags. This link is the third step combining narrative education: Practice.	Professional quality: Through the integration of theory and practice, nursing students’ initiative, enthusiasm, and creativity were enhanced, while cultivating professional qualities such as ethical self-discipline, compassion, and benevolence. Occupational safety: Students were guided to strengthen their awareness of occupational safety during skill-based training. Legal literacy: Training reinforced students’ familiarity with relevant medical and nursing laws and regulations, deepened their understanding of technical standards, and encouraged the development of a legal mindset and rule-of-law awareness. Value shaping: Nursing students were guided to uphold professional morality by respecting patients, cherishing life, and treating all individuals with equality and dignity.
Post-test (P)	Wednesday of week 3	Post-test questions were released on the XUEXITONG platform, consisting of five multiple-choice items: (1) What position should a patient assume at the initiation of artificial anus opening after radical rectal cancer resection? (2) Which nursing practice is incorrect for patients with an artificial anus after surgery? (3) A 52-year-old female patient presents with long-term hematochezia of unknown cause and progressive emaciation. Colonoscopy is indicated for further diagnosis. What should be the nurse’s first action before the examination? (4) What is the purpose of administering oral metronidazole to rectal cancer patients 3 days before surgery as prescribed? (5) When collecting the medical history of patients with colon cancer, what should be the key focus of inquiry? In addition, online Q&A sessions and teacher-student interactions were conducted to consolidate learning.	Humanities literacy: Test questions on nursing measures for colorectal cancer patients were designed to cultivate students’ humanistic qualities, including respect for patients, equal treatment, protection of privacy, and the development of empathy. Professional quality: Nursing students were guided to uphold rigorous and pragmatic professional ethics, fostering a spirit of dedication and commitment to their profession.
Summary (S)	Wednesday of week 3	Once again, the department’s chief instructor integrated the narrative materials to generalize, analyze, and summarize the theoretical and practical knowledge of colorectal cancer, addressing the main points, key concepts, and learning challenges. Questions with a high error rate from the pre- and post-tests were reviewed and analyzed. The instructor emphasized the importance of health education and follow-up guidance for colorectal cancer patients, underscoring that only by embracing the concept of a *“healthy life”* can the national call for *“Healthy China”* be effectively answered. After class, supplementary materials, including courseware, videos, recent literature, and other review and tutorial resources, were released on the XUEXITONG platform.	Political identity: Emphasis was placed on the concepts of holistic health, comprehensive well-being, and prevention first, aligning with the “Healthy China 2030” blueprint and reflecting the characteristics of the nursing profession. Scientific spirit: Through the induction, analysis, and summarization of key knowledge points, nursing students cultivated a scientific spirit characterized by diligence in study, pursuit of truth, and pragmatism.
Participatory (P)	Thursday and Friday of week 3	The same as those on Tuesday and Wednesday	The same as those on Tuesday and Wednesday
Summary (S)	Friday of week 3	The department’s chief instructor selected cases from the narrative material library and organized nursing students for ward rounds. One case involved the nursing care of a rectal cancer patient with postoperative stoma stenosis. After completing the ward round, students engaged in open discussion. Teachers participated in these group discussions, providing guidance and highlighting key aspects of ward-round practice. Group representatives then presented their reports, followed by the instructors’ summary of the week’s nursing practice.	Scientific spirit: Nursing ward rounds encouraged students to shift from passive knowledge acquisition to active exploration and inquiry. Professional quality: Ward rounds helped nursing students cultivate the professional ethos of healthcare providers, dedicated to saving lives, healing the wounded, and demonstrating boundless compassion.
Summary (S)	Saturday of week 3	Nursing students reflected on what they had learned, observed, and practiced during the week by keeping reflection diaries, recording and consolidating their experiences in writing. They shared their insights, feelings, and discussions on the XUEXITONG platform, enabling peer learning and fostering resonance among students. This link is the fourth step combining narrative education: Sentiment guidance.	Humanities literacy, scientific thinking, and evidence-based practice: By keeping reflection diaries, nursing students refined their emotional expression, internalized professional knowledge and humanistic qualities, strengthened their sense of social responsibility and professional ethics, and cultivated scientific thinking along with evidence-based practice.
Expansion	Sunday in week 3	Content related to specialist nurses, enterostomal therapists, was released on the XUEXITONG platform.	Scientific spirit: Students’ knowledge and skills were broadened, helping them recognize that learning is a lifelong process while fostering a spirit of openness and exploration. Professional quality: Nursing students were guided to develop innovative thinking, a pioneering mindset, and a commitment to professional excellence.

#### 2.2.3 Control of teaching time and resource balance between groups

Teaching time: The clinical internship for both groups of nursing students lasted 4 weeks, with a total weekly teaching duration of 32 h. This included 8 h of offline theoretical lectures, 20 h of clinical teaching and practical operations, and 4 h of assessment and Q&A. For the experimental group, the BOPPPS components and narrative education were integrated into the existing 32-h schedule. Online activities on the XUEXITONG platform, such as pre-class preparation, quizzes, and material review, were completed during the corresponding offline teaching periods. These activities did not require additional extracurricular time or increase the total teaching hours.

Learning resources: Both groups of nursing students used the same clinical teaching resources in the gastrointestinal surgery department. These included standardized theoretical lecture presentations, clinical operation demonstration videos, operation models, and clinical case materials. The narrative materials and supplementary resources uploaded for the experimental group on the XUEXITONG platform were derived from the department’s uniformly organized teaching materials. Control group students had access to the same content through offline departmental resources. The experimental group benefited primarily from the integrated and more accessible presentation of these materials.

### 2.3 Evaluation method

**Figure 2:**
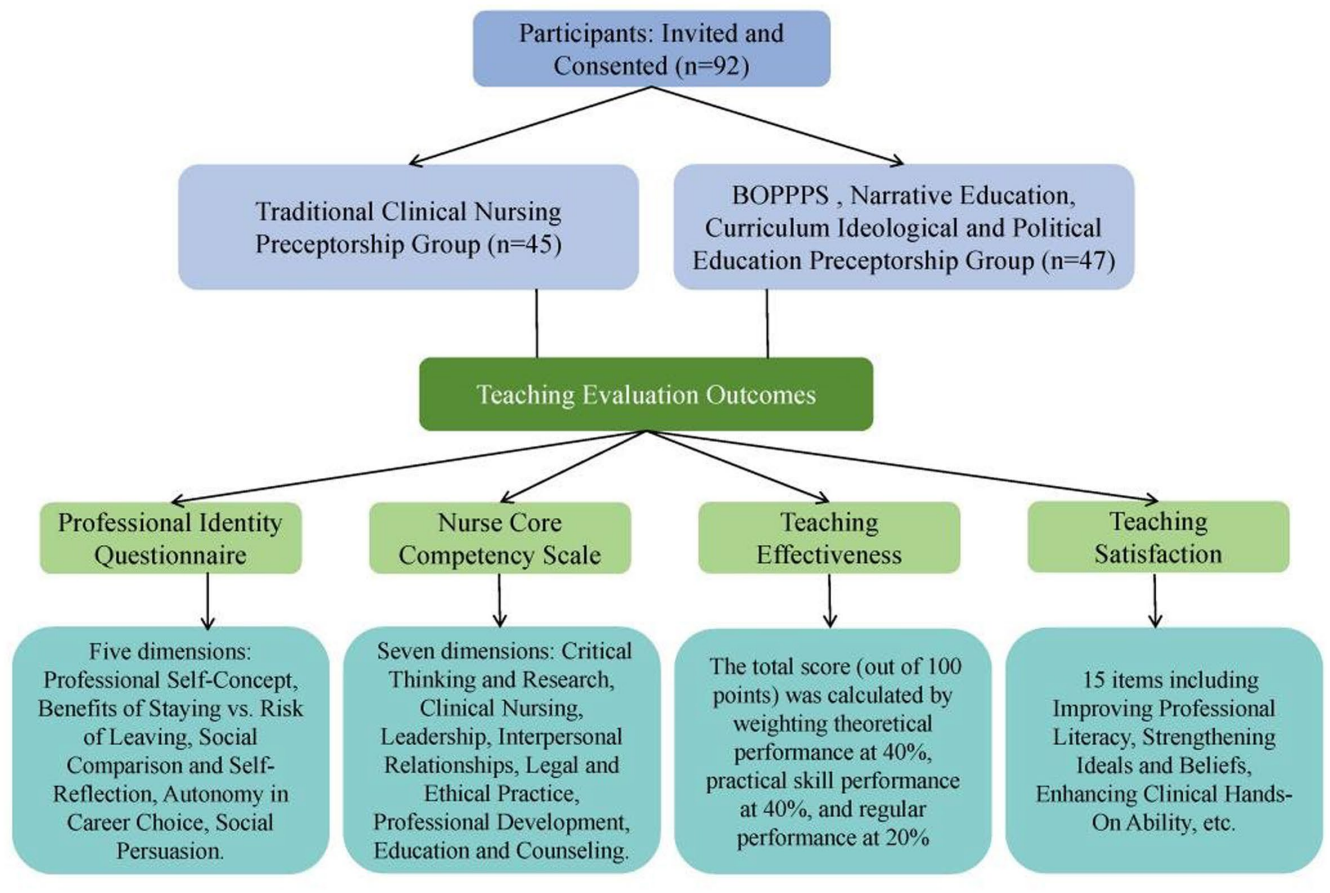
Overview of the research design and effect evaluation.

The study design and evaluation methods are illustrated in Figure 2. The teaching effect on nursing students was assessed by simulating and evaluating their clinical practice abilities, combined with a questionnaire survey conducted before they completed their rotation. The evaluation timing, methods, and standards were consistent for both groups.

#### 2.3.1 Professional identity

The Professional Identity Questionnaire for Nursing Students (PIQNS), developed by Hao (23), was used to assess the professional identity of nursing students. The questionnaire includes 17 items across five dimensions: professional self-concept (6 items), retention benefits and resignation risk (4 items), social comparison and self-reflection (3 items), autonomy in career choice (2 items), and social persuasion (2 items). A Likert scale was used, with responses ranging from “very inconsistent” to “very consistent,” scored 1–5, respectively; the 12th item was reverse-scored. Higher scores indicate a higher level of professional identity. In this study, the questionnaire demonstrated good reliability, with a Cronbach’s *α* coefficient of 0.823.

#### 2.3.2 Core competence

The Competency Inventory for Registered Nurses (CIRN), developed by Liu et al. (24), was used to assess the core competence of nursing students. The scale includes 58 items across seven dimensions: critical thinking and scientific research (10 items), clinical nursing (9 items), leadership (10 items), interpersonal relationships (8 items), legal and ethical practice (8 items), career development (6 items), and education and consultation (7 items). A Likert scale was used, with responses ranging from “not capable” to “very capable,” scored 0–4, respectively. Higher scores indicate greater and more comprehensive core competence. In this study, the scale demonstrated high reliability, with a Cronbach’s *α* coefficient of 0.894.

#### 2.3.3 Teaching effect

In the 4th week of the internship, students completed end-of-rotation examinations in both theoretical knowledge and skills operations. The total score was calculated as: Theory Score × 40% + Skills Operation Score *40% + Usual Performance Score *20%. The theory exam was closed-book and consisted of true/false questions (20 points), multiple-choice questions (40 points), multiple-answer questions (20 points), and case analysis questions (20 points). For the skills assessment, instructors selected one basic nursing operation (e.g., intravenous infusion, indwelling catheterization, intramuscular injection) and one specialized nursing operation (e.g., replacing a gastrointestinal decompressor, replacing a drainage bag, replacing an ostomy bag) for each student, without prior notice. Each operation was scored out of 100, and the average of the two scores formed the skills operation score. The theoretical and skills assessment content was identical for both groups. Usual performance was evaluated comprehensively by clinical teaching instructors based on students’ overall performance during practice, with a maximum score of 100.

The end-of-rotation theory exam and skills operation assessment were conducted using a strict double-blind procedure. (1) Blinding of assessors: All judges were senior nursing staff from the department who were not involved in the daily teaching of either group. They were unaware of students’ group assignments and knew only the assessment numbers. (2) Blinding of students: Students were not informed of the identities of the assessment judges or the specific scoring criteria. Only the core content and time requirements of the assessment were disclosed in advance. (3) Blinding of the scoring process: Theory exams were graded using sealed papers in a double-check system. Papers were identified only by assessment numbers, without personal identifiers. During skills operation assessments, students wore uniform numbered badges, and judges scored based solely on the quantitative scoring scale. After scoring, designated personnel verified the numbers against students’ personal information.

Standardized assessment of comprehensive total scores. (1) Theory scores: Prior to the study, the gastrointestinal surgery nursing teaching team, in collaboration with the department’s quality control team, developed the exam questions and scoring standards. These materials were uniformly sealed and stored. Both groups of students used the same exam questions, ensuring full consistency in content, scoring criteria, and assessment procedures. (2) Skills operations: (i) Assessment scenarios, equipment, models, and consumables were standardized and conducted in a simulation ward. (ii) Standardized operational procedures were established, such as the eight-step process for ostomy bag replacement, clarifying key steps and sequence. (iii) A quantitative scoring scale was applied, breaking each operation into 10–15 key scoring points with defined scores and deduction criteria. (iv) The assessment process was standardized. Students were informed of time limits in advance. During assessments, judges observed and recorded performance only, without providing prompts or guidance. (3) Usual performance: (i) Unified evaluation dimensions were set, including internship attitude (20 points), operational standardization (30 points), communication ability (20 points), teamwork (15 points), and emergency response (15 points), with corresponding sub-indicators. (ii) Detailed evaluation guidelines were developed, specifying deduction criteria for each sub-indicator. Instructors received unified training before applying the quantitative scoring system. (iii) Instructors conducted weekly evaluations, recording key performance events as a basis for scoring. (iv) After the internship, the teaching team cross-reviewed all scores. Differences exceeding five points were discussed in combination with performance records to reach a consensus, ensuring objectivity.

To ensure consistency in assessment scoring, inter-rater reliability was verified before the formal evaluation. Three judges conducted a simulated assessment on 10 randomly selected nursing students, completing blind scoring independently. The Intraclass Correlation Coefficient (ICC) was used to assess inter-rater consistency. The results were as follows: theory exam scoring ICC = 0.92 (95% CI: 0.85–0.96), skills operation assessment ICC = 0.90 (95% CI: 0.83–0.95), and usual performance scoring ICC = 0.88 (95% CI: 0.80–0.94). All values indicate good inter-rater consistency (ICC > 0.75). These findings suggest that the assessment scoring system was reliable and minimized the potential impact of evaluator bias on the study results.

#### 2.3.4 Teaching satisfaction

The teaching satisfaction evaluation questionnaire, designed by the research team, was used to assess students’ satisfaction with the teaching program. It comprised three dimensions: Value and Professional Literacy (5 items), Cognitive Competence and Ability (7 items), and Teaching Evaluation (3 items), for a total of 15 items. The questionnaire was reviewed and refined by five experts, yielding an item-level content validity index (I-CVI) of 0.80–1.00 and a scale-level content validity index (S-CVI) of 0.92. Following a pretest with 20 nursing students and subsequent expression revisions, the questionnaire was formally administered. Reliability analysis showed an overall Cronbach’s *α* of 0.913, with dimension-specific coefficients ranging from 0.763 to 0.897. Exploratory factor analysis (EFA) identified three common factors, explaining a cumulative variance of 67.865%, indicating satisfactory psychometric properties. A Likert scale was used, with scores ranging from 1 (“unsatisfied”) to 5 (“very satisfied”); higher scores indicated greater teaching satisfaction. Questionnaires were distributed uniformly at the end of the students’ clinical practice, completed anonymously, and collected on the spot. A total of 92 valid questionnaires were returned, yielding an effective recovery rate of 100%.

### 2.4 Pre-experimental power analysis and sample size estimation

This study compared measurement data from two independent samples. An *a priori* power analysis and sample size estimation were conducted using G*Power 3.1.9.7 software. Based on similar previous nursing teaching interventions (25), the primary outcome was set as the total score of the nursing students’ professional identity questionnaire. The between-group mean difference was assumed to be 7.67, with a pooled standard deviation of 1.37. With a significance level of *α* = 0.05 (two-tailed) and a desired power of 1 − *β* = 0.90 (the recommended standard for clinical research), the minimum required sample size was calculated as 32 participants per group. To account for potential loss to follow-up or incomplete assessments, the sample size was increased by 20%, yielding a minimum requirement of 39 participants per group. The final study included 45 participants in the control group and 47 in the experimental group, both exceeding the estimated minimum and ensuring adequate statistical power to support the reliability of the results.

### 2.5 Statistical analysis

Statistical analyses were performed using SPSS 27.0. Measurement data are presented as mean ± standard deviation (x̄ ± s). Between-group comparisons were performed using the independent-samples t-test. Normality was assessed with the Shapiro–Wilk test, homogeneity of variance with Levene’s test, and the consistency of inter-group change trends with the parallelism test. Post-teaching scores were compared between groups using a one-way analysis of covariance (ANCOVA), with pre-teaching scores included as a covariate. The Bonferroni method was applied for multiple comparison correction, and partial *η*^2^ and Cohen’s d values were reported to indicate effect sizes. Enumeration data are presented as frequencies and percentages (%), and between-group differences were assessed using the *χ*^2^ test. Differences were considered statistically significant at *p* < 0.05.

## 3 Results

### 3.1 Comparison of scores of professional identity questionnaire between two groups of nursing students

Tests for normality, homogeneity of variance, and parallelism all satisfied the assumptions for parametric tests (all *p* > 0.05). ANCOVA analyses, following Bonferroni correction for multiple comparisons, demonstrated that the experimental group achieved significantly higher scores on all dimensions and the total score of the professional identity questionnaire than the control group (all *p* < 0.001). Effect size analysis revealed partial *η*^2^ values ranging from 0.462 to 0.891, indicating large effect sizes. Detailed results are presented in Tables 2, 3.

**Table 2:** Comparison of scores of professional identity questionnaire between the two groups of nursing students (scores, $\bar{x}\pm s$ ).

Item	Pre-intervention		*t*-value	*p*-value	Post-intervention		*t*-value	*p*-value
	Control group (*n* = 45)	Experimental group (*n* = 47)			Control group (*n* = 45)	Experimental group (*n* = 47)		
Total score	60.29 ± 2.11	60.26 ± 1.59	−0.086	0.931	62.82 ± 1.59	70.49 ± 1.14	26.732	<0.001
Professional self-concept	21.09 ± 1.31	21.02 ± 0.94	0.285	0.776	22.02 ± 0.87	23.83 ± 0.79	10.474	<0.001
Job retention benefits and turnover risk	14.22 ± 0.82	14.23 ± 0.73	0.073	0.942	15.04 ± 0.74	16.21 ± 0.62	8.221	<0.001
Social comparison and self-reflection	10.91 ± 0.63	10.91 ± 0.62	0.029	0.977	11.11 ± 0.57	12.81 ± 0.61	13.709	<0.001
Autonomy in career choice	7.00 ± 0.63	6.98 ± 0.66	0.166	0.868	7.31 ± 0.56	8.85 ± 0.36	15.679	<0.001
Social persuasion	7.09 ± 0.62	7.09 ± 0.56	−0.013	0.976	7.33 ± 0.56	8.79 ± 0.41	14.048	<0.001

**Table 3:** ANCOVA of post-test scores for professional identity questionnaire between the two groups of nursing students.

Variable	Group	*n*	SD	Mean	SE	F	P	η^2^
Professional self-concept	Control grouproup	45	0.866	22.020a	0.124	109.655	<0.001	0.552
	Experimental group	47	0.789	23.832a	0.121			
Job retention benefits and turnover risk	Control group	45	0.737	15.046a	0.095	76.405	<0.001	0.462
	Experimental group	47	0.623	16.211a	0.093			
Social comparison and self-reflection	Control group	45	0.573	11.112a	0.070	299.836	<0.001	0.771
	Experimental group	47	0.613	12.807a	0.068			
Autonomy in career choice	Control group	45	0.557	7.313a	0.067	265.634	<0.001	0.749
	Experimental group	47	0.360	8.849a	0.066			
Social persuasion	Control group	45	0.564	7.332a	0.063	270.322	<0.001	0.752
	Experimental group	47	0.414	8.788a	0.062			
Professional identity total score	Control group	45	1.585	62.820a	0.203	730.522	<0.001	0.891
	Experimental group	47	1.140	70.492a	0.199			

### 3.2 Comparison of scores of core competence scale between two groups of nursing students

Tests for normality, homogeneity of variance, and parallelism confirmed that the assumptions for parametric analyses were met (all *p* > 0.05). ANCOVA, with Bonferroni correction for multiple comparisons, showed that the experimental group scored higher than the control group on all dimensions and the total score of the Core Competency Scale for nursing students (all *p* < 0.001). Effect size analysis yielded partial *η*^2^ values ranging from 0.657 to 0.970, indicating large effects. Detailed results are presented in Tables 4, 5.

**Table 4:** Comparison of scores of core competence scale between two groups of nursing students (Scores, $\bar{x}\pm s$ ).

Item	Pre-intervention		*t*-value	*p*-value	Post-intervention		*t*-value	*p*-value
	Control group (*n* = 45)	Experimental group (*n* = 47)			Control group (*n* = 45)	Experimental group (*n* = 47)		
Total score	123.78 ± 2.71	124.23 ± 3.01	0.763	0.448	134.53 ± 2.74	164.60 ± 2.64	53.614	< 0.001
Critical thinking and research	23.02 ± 0.62	23.09 ± 1.35	0.289	0.773	23.69 ± 1.15	27.11 ± 1.13	14.426	< 0.001
Clinical nursing	21.33 ± 1.17	21.32 ± 1.14	−0.059	0.953	23.53 ± 1.41	29.49 ± 1.04	23.151	< 0.001
Leadership	20.18 ± 1.37	20.40 ± 1.73	0.695	0.489	22.91 ± 1.10	26.13 ± 1.30	12.789	< 0.001
Interpersonal relationships	18.91 ± 0.63	18.98 ± 0.87	0.424	0.673	20.40 ± 1.23	24.34 ± 1.40	14.289	< 0.001
Legal and ethical practice	17.07 ± 1.42	17.06 ± 1.28	−0.010	0.992	18.36 ± 1.19	22.83 ± 1.11	18.660	< 0.001
Professional development	11.93 ± 0.58	12.17 ± 0.60	0.992	0.258	13.00 ± 0.67	18.62 ± 1.15	28.677	< 0.001
Education and counseling	11.33 ± 0.95	11.21 ± 1.02	−0.585	0.560	12.64 ± 0.68	16.09 ± 0.65	24.754	< 0.001

**Table 5:** ANCOVA of Post-test Scores for the Core Competence Scale Between the Two Groups of Nursing Students.

Variable	Group	*n*	SD	Mean	SE	*F*	*p*	*η* ^2^
Critical thinking and research	Control group	45	1.145	23.683a	0.168	212.960	<0.001	0.705
	Experimental group	47	1.127	27.112a	0.164			
Clinical nursing	Control group	45	1.408	23.531a	0.178	569.915	<0.001	0.865
	Experimental group	47	1.040	29.491a	0.175			
Leadership	control group	45	1.104	22.938a	0.173	170.743	<0.001	0.657
	Experimental group	47	1.296	26.102a	0.169			
Interpersonal relationships	Control group	45	1.232	20.396a	0.198	203.190	<0.001	0.695
	Experimental group	47	1.403	24.344a	0.194			
Legal and ethical practice	Control group	45	1.190	18.355a	0.146	481.715	<0.001	0.844
	Experimental group	47	1.110	22.830a	0.143			
Professional development	Control group	45	0.674	13.012a	0.144	761.080	<0.001	0.895
	Experimental group	47	1.153	18.606a	0.140			
Education and counseling	Control group	45	0.679	12.634a	0.097	649.454	<0.001	0.879
	Experimental group	47	0.654	16.095a	0.095			
Core competence total score	Control group	45	2.735	134.548a	0.403	2831.691	<0.001	0.970
	Experimental group	47	2.643	164.582a	0.394			

### 3.3 Comparison of teaching effect on two groups of nursing students

After the intervention, the nursing students in the experimental group achieved higher theoretical scores, operation scores, usual performance scores, and total scores compared with the control group, with the differences being statistically significant (*p* < 0.001). Cohen’s d values ranged from 0.86 to 2.00, indicating large effect sizes. Detailed results are shown in Table 6.

**Table 6:** Comparison of teaching effect on two groups of nursing students (Scores, $\bar{x}\pm s$ ).

Item	Control group (*n* = 45)	Experimental group (*n* = 47)	*t*-value	*p*-value	Cohen’s d
Theoretical score	87.71 ± 1.94	92.13 ± 2.18	10.245	<0.001	2.067
Clinical skills score	88.24 ± 1.82	93.02 ± 1.55	13.546	<0.001	1.691
Routine performance score	94.98 ± 0.87	97.11 ± 0.87	11.790	<0.001	0.866
Total score	89.38 ± 1.15	93.48 ± 0.99	18.401	<0.001	1.069

### 3.4 Evaluation of teaching satisfaction of two groups of nursing students with different teaching methods

The nursing students in the experimental group scored higher than those in the control group on each item of the teaching satisfaction evaluation questionnaire, with the differences being statistically significant (*p* < 0.001). Cohen’s d values ranged from 0.465 to 0.634, indicating moderate to large effect sizes. Detailed results are presented in Table 7.

**Table 7:** Evaluation of teaching satisfaction of two groups of nursing students with different teaching methods (Scores, $\bar{x}\pm s$ ).

Item	Control group (*n* = 45)	Experimental group (*n* = 47)	*t*-value	*p*-value	Cohen’s d
Strengthening ideals and beliefs	3.02 ± 0.66	4.55 ± 0.50	12.590	< 0.001	0.583
Stimulating patriotism and family affection	3.02 ± 0.50	4.60 ± 0.50	15.155	< 0.001	0.498
Enhancing legal awareness	3.24 ± 0.48	4.38 ± 0.49	11.190	< 0.001	0.488
Cultivating scientific spirit	3.09 ± 0.56	4.45 ± 0.50	12.262	< 0.001	0.530
Improving professional literacy	3.27 ± 0.54	4.60 ± 0.54	11.829	< 0.001	0.539
Enhancing learning initiative	3.09 ± 0.56	4.40 ± 0.50	11.975	< 0.001	0.527
Increasing learning interest	3.00 ± 0.56	4.45 ± 0.50	12.970	< 0.001	0.534
Promoting clinical hands-on ability	3.40 ± 0.54	4.43 ± 0.50	9.465	< 0.001	0.520
Enhancing comprehensive analysis ability	2.96 ± 0.67	4.19 ± 0.47	9.932	< 0.001	0.576
Improving problem-solving ability	2.98 ± 0.69	4.23 ± 0.56	9.604	< 0.001	0.627
Developing clinical judgment	3.07 ± 0.50	4.32 ± 0.63	10.632	< 0.001	0.568
Fostering teamwork spirit	3.27 ± 0.65	4.28 ± 0.62	7.635	< 0.001	0.634
Satisfaction with teaching effect	3.69 ± 0.47	4.62 ± 0.49	9.268	< 0.001	0.480
Satisfaction with teaching management	3.78 ± 0.42	4.47 ± 0.50	7.142	< 0.001	0.465
Satisfaction with teaching methods	3.29 ± 0.46	4.62 ± 0.49	13.392	< 0.001	0.476

## 4 Discussion

### 4.1 Positive association of the BOPPPS-narrative model integrating curriculum ideology and politics with nursing students’ professional identity

The results of this study indicated that the experimental group scored higher than the control group across all dimensions and in the total score of professional identity (*p* < 0.001). The intervention demonstrated a large effect size, suggesting practical significance in supporting the development of professional identity among nursing students, consistent with findings from related research (25, 26). From the perspective of teaching implementation, the narrative education method used authentic narrative materials to create emotionally relevant scenarios related to gastrointestinal surgery (15), including the care experiences of ostomy patients and the challenges of postoperative recovery. This approach may facilitate students’ understanding of patients’ experiences and needs, gradually fostering empathy and humanistic care abilities (27), and potentially supporting their emotional alignment with the value of the nursing profession. Within the participatory learning component of the BOPPPS model, teachers organized students for group discussions, analysis of narrative cases, and clinical practice activities. These experiences may have progressively deepened students’ understanding of the essence of the nursing profession (3). The summary component, which guided students to write reflective journals, offered an opportunity for self-examination of professional cognition and reinforcement of professional beliefs, potentially transforming external professional guidance into internalized professional identity (28). Additionally, curriculum ideology and politics (29), by integrating value orientations such as “respect for life” and “dedication” into teaching, may help students gradually establish value-based identification with the nursing profession while simultaneously mastering professional skills.

It should be noted that the development of professional identity is a long-term process. This study reflects only short-term changes observed during the internship period. Professional identity is influenced by multiple factors, including individual emotional experiences (30), clinical practice feedback (31), and career planning (32), and cannot be attributed solely to the intervention. However, given the comparable baseline characteristics between the two groups and the rigorous quality control measures employed, it is plausible that this teaching model may contribute positively to the enhancement of professional identity.

### 4.2 Association of the BOPPPS-narrative model integrating curriculum ideology and politics with the enhancement of nursing students’ core competence

The results of this study indicated that the experimental group scored higher than the control group across all dimensions and in the total score of core competence (*p* < 0.001). The intervention demonstrated a large effect size, suggesting that this teaching model has practical value in supporting the development of nursing students’ core competence, consistent with previous studies in China (33, 34). Core competence is essential for nursing students to perform effectively in clinical practice, encompassing areas such as critical thinking (35), clinical nursing (36), and professional development (37). Participatory learning, a core component of the BOPPPS model, involved presenting challenging clinical nursing problems and organizing team-based collaborative inquiry. This approach may encourage students to actively explore and develop clinical reasoning for problem-solving (38), potentially supporting improvements in clinical nursing abilities, including operational skills and innovative practice (39). Post-class supplementary materials provided opportunities for deeper engagement with professional development, which may foster students’ scientific inquiry and exploratory mindset. During narrative education, students analyzed and reflected on narrative cases, followed by practical operations and nursing rounds under teacher guidance. These activities may help students systematically organize key aspects of nursing care and clinical decision-making (40), laying a foundation for proposing innovative solutions and further developing critical thinking, teamwork, and communication skills. Furthermore, the teaching model’s integration of curriculum ideology and politics guided students to consider ethical and moral perspectives when addressing clinical issues. This approach may encourage greater caution and thoughtfulness regarding patient safety and nursing quality in practice (41), thereby contributing to the development of rigorous professional competence.

It is important to recognize that the development of core competence reflects the combined influence of multiple factors, including knowledge accumulation (42), skill training (43), and the development of thinking abilities (44). Although the observed advantage in the experimental group is likely related to the teaching model intervention, it may also be affected by other factors, such as students’ learning initiative and clinical practice opportunities. Therefore, the findings of this study suggest a significant association between the combined teaching model and the enhancement of nursing students’ core competence, rather than establishing a definitive causal relationship.

### 4.3 Association of the BOPPPS-narrative model integrating curriculum ideology and politics with improved clinical teaching outcomes

In this study, the theoretical, operational, and usual performance scores, as well as the total scores of nursing students in the experimental group, were significantly higher than those in the control group (*p* < 0.001). Large effect sizes were observed for the intervention. These results suggest that the teaching model may support improvements in the effectiveness of clinical teaching, consistent with previous findings (2, 38). The BOPPPS teaching model, by establishing clear learning objectives and structured teaching components, guided students in identifying key points and difficulties during the internship. This targeted approach may facilitate more effective mastery of professional knowledge. The pre-test helped assess students’ understanding of basic concepts and allowed for dynamic adjustment of teaching strategies (45). During participatory learning, students actively constructed their own knowledge through practice, discussion, and reflection, potentially promoting the development of autonomous learning abilities (46). The post-test provided an assessment of internship outcomes, highlighted areas for improvement, and may have motivated students for subsequent learning (19). Activities involving summarization and reflection (47) reinforced knowledge retention and may have contributed to improved teaching effectiveness. The incorporation of narrative education further enhanced teaching engagement and practicality by using real cases to concretize abstract medical concepts, potentially reducing difficulties in comprehension (48). The integration of curriculum ideology and politics elements may have helped students recognize their professional value and responsibilities, stimulated learning interest and initiative, and provided additional motivation and meaning in the learning process (29). As a result, students may have been more focused and diligent during the internship. Additionally, the technical support provided by the XUEXITONG platform optimized the presentation of teaching resources and facilitated interactive learning, further supporting improvements in teaching effectiveness.

It should be emphasized that improvements in teaching effectiveness reflect the combined influence of multiple factors, including optimization of the teaching model (49), standardization of teaching implementation (50), and active student engagement (17). In addition, instructors’ accumulated teaching experience may contribute modestly to positive outcomes (51). Therefore, differences in teaching effectiveness should be interpreted as the result of the integrated effects of the teaching model and other contributing factors, with the model serving as a central driver rather than the sole determinant.

### 4.4 Positive association of the BOPPPS-narrative model integrating curriculum ideology and politics with teaching satisfaction

The results of this study indicated that nursing students in the experimental group scored significantly higher than those in the control group on all items of teaching satisfaction (*p* < 0.001). Moderate to large effect sizes were observed for the intervention. These findings suggest that the teaching model may support improvements in teaching satisfaction, consistent with previous studies (25, 34). Teaching satisfaction (52) reflects students’ recognition of the teaching content and methods and can influence their learning outcomes and professional development. The six diverse components of the BOPPPS model enriched the teaching process and made it more interactive. The student-centered design (53) actively engaged students, potentially facilitating knowledge internalization. The model encouraged students to participate and express their opinions, allowing them to experience greater enjoyment and a sense of achievement, which may contribute to higher teaching satisfaction (54). Narrative education, through real clinical cases or related videos, captured students’ attention and stimulated learning interest (55). Group discussions and reflective journals provided opportunities for students to share their learning experiences and insights. This interactive approach may enhance learning motivation and also support the development of teamwork and critical thinking skills (56). Furthermore, the integration of curriculum ideology and politics (29) enabled students to recognize the social value and sense of mission associated with the nursing profession, further enhancing their engagement with the teaching content and methods.

It should be noted that teaching satisfaction is influenced by multiple factors, including teaching content (57), teaching methods (58), and teacher-student interaction (50). Although the higher satisfaction observed in the experimental group is likely related to the integrated teaching model, it may also be affected by factors such as students’ novelty toward the model, instructors’ engagement, and individual learning experiences. Therefore, these results primarily reflect students’ subjective recognition of the combined teaching model, and their objective relationship with improvements in teaching quality should be interpreted alongside other evaluation indicators.

### 4.5 Study limitations

#### 4.5.1 Research design level

This study employed a quasi-experimental sequential cohort design with a historical control group based on internship batches. Although confounding factors were minimized as much as possible through baseline equivalence testing, standardized teaching resources, uniform operational procedures, assessor blinding, and inter-rater reliability checks, potential biases arising from non-random assignment, such as temporal effects and batch differences, could not be fully eliminated. Therefore, this study only indicates an association between the teaching model and the outcome measures and does not establish a definitive causal relationship. Future research may adopt a multicenter randomized controlled design (59) to further improve the internal validity and causal interpretability of the findings.

#### 4.5.2 Intervention implementation level

The overall effects observed in this study are the result of a comprehensive intervention. Because no control subgroups for individual components were included, it was not possible to isolate the independent effects, interaction effects, or core driving components of the BOPPPS teaching model, narrative education, curriculum ideology and politics, and the XUEXITONG platform. In addition, the experiential differences between interactive learning and digital resource presentation in the experimental group versus traditional teaching in the control group may have contributed to the observed outcomes. Future research could adopt a factorial design (60), incorporating intervention control subgroups with different combinations of components to examine the effects of each element and identify the optimal combination. Qualitative methods, such as semi-structured student interviews and analysis of teachers’ instructional logs, could also be employed to clarify the contribution pathways of each component to teaching effectiveness.

#### 4.5.3 Fidelity verification level

This study did not include a protocol to verify intervention fidelity. Although instructors received standardized training and teaching requirements were clearly defined, the degree of implementation of teaching components was not quantitatively assessed through methods such as classroom observations, teaching record reviews, or teacher interviews. As a result, variations in teaching details and interaction depth among instructors may have occurred, introducing implementation bias and potentially influencing the outcomes of the intervention. Future research should establish a comprehensive fidelity verification system (61), which could include the following steps: First, develop a standardized checklist specifying the key points and evaluation criteria for each BOPPPS component, the four steps of narrative education, and the integration of ideological and political elements. Second, have independent researchers randomly sample over 30% of teaching sessions and score them using the checklist through on-site observation or video review. Third, regularly collect instructors’ teaching logs and students’ learning records to monitor implementation consistency. Finally, provide secondary training and targeted guidance for instructors whose implementation does not meet the standards, ensuring adherence to the planned teaching protocol.

## 5 Conclusion

This study implemented a teaching model that combines the BOPPPS framework with narrative education, integrated with curriculum ideology and politics, in clinical nursing teaching for gastrointestinal surgery. The BOPPPS model provided a structured teaching process, while narrative education enriched the teaching content. Curriculum ideology and politics were incorporated throughout all teaching components, and an information platform facilitated the coordinated integration of these elements. Together, these components formed a clinical nursing teaching pathway. The implementation of this pathway was associated with integrated knowledge acquisition, skills development, and value guidance. Although this study has certain limitations, it offers a practical approach to clinical nursing education. Future research could address these limitations, optimize the model, and explore its generalizability across additional application scenarios.

## Data Availability

The original contributions presented in the study are included in the article/supplementary material, further inquiries can be directed to the corresponding author/s.
